# Exosomal microRNA-15a from mesenchymal stem cells impedes hepatocellular carcinoma progression via downregulation of SALL4

**DOI:** 10.1038/s41420-021-00611-z

**Published:** 2021-08-28

**Authors:** Yu-Shui Ma, Ji-Bin Liu, Lan Lin, Hui Zhang, Jian-Jun Wu, Yi Shi, Cheng-You Jia, Dan-Dan Zhang, Fei Yu, Hui-Min Wang, Yu-Zhen Yin, Xiao-Hui Jiang, Pei-Yao Wang, Lin-Lin Tian, Ping-Sheng Cao, Xu-Ming Wu, Hai-Min Lu, Li-Peng Gu, Jia-Jia Zhang, Gu-Jun Cong, Pei Luo, Xiao-Ming Zhong, Bo Cai, Min-Xin Shi, Su-Qing Zhang, Liu Li, Wen-Jie Zhang, Yu Liu, Zhi-Zhen Li, Ting-Miao Wu, Zhi-Jun Wu, Gao-Ren Wang, Zhong-Wei Lv, Chang-Chun Ling, Kai-Jian Chu, Da Fu

**Affiliations:** 1 Central Laboratory for Medical Research, Shanghai Tenth People’s Hospital, Tongji University School of Medicine, 200072 Shanghai, China; 2 Department of Laboratory Medicine, Nantong Tumor Hospital, Tumor Hospital Affiliated of Nantong University, 226631 Nantong, China; 3 International Cooperation Laboratory on Signal Transduction, Eastern Hepatobiliary Surgery Hospital/Institute, National Center for Liver Cancer, the Second Military Medical University, 200433 Shanghai, China; 4 Nantong Haimen Yuelai Health Centre, 226100 Haimen, China; 5 Department of Nuclear Medicine, Shanghai Tenth People’s Hospital, Tongji University School of Medicine, 200072 Shanghai, China; 6 Department of Pathology, Shihezi University School of Medicine, the First Affiliated Hospital of Shihezi University School of Medicine, 832003 Shihezi, Xinjiang China; 7 General Surgery, Nantong Tumor Hospital, 226631 Nantong, China; 8 Department of Chronic Diseases, Nantong Center for Disease Control and Prevention, 226007 Nantong, China; 9 Department of Thoracic Surgery, Nantong Tumor Hospital, 226631 Nantong, China; 10 Health Examination Centers, Nantong Tumor Hospital, 226631 Nantong, China; 11 Department of Tumor Radiotherapy, Jiangxi Provincial Tumor Hospital, 330029 Nanchang, China; 12 Department of Hepatobiliary Surgery, Nantong Tumor Hospital, 226631 Nantong, China; 13 Department of Biliary Tract Surgery I, Shanghai Eastern Hepatobiliary Surgery Hospital, 200438 Shanghai, China; 14 Department of Radiology, The Fourth Affiliated Hospital of Anhui Medical University, 230012 Hefei, China; 15 Department of Radiotherapy, Nantong Tumor Hospital, 226631 Nantong, China; 16 Department of General Surgery, The Affiliated Hospital of Nantong University, 226001 Nantong, China

**Keywords:** Cancer, Cancer stem cells

## Abstract

Hepatocellular carcinoma (HCC) is a heterogeneous tumor with an increased incidence worldwide accompanied by high mortality and dismal prognosis. Emerging evidence indicates that mesenchymal stem cells (MSCs)-derived exosomes possess protective effects against various human diseases by transporting microRNAs (miRNAs or miRs). We aimed to explore the role of exosomal miR-15a derived from MSCs and its related mechanisms in HCC. Exosomes were isolated from transduced MSCs and co-incubated with Hep3B and Huh7 cells. miR-15a expression was examined by RT-qPCR in HCC cells, MSCs, and secreted exosomes. CCK-8, transwell, and flow cytometry were used to detect the effects of miR-15a or spalt-like transcription factor 4 (SALL4) on cell proliferative, migrating, invasive, and apoptotic properties. A dual-luciferase reporter gene assay was performed to validate the predicted targeting relationship of miR-15a with SALL4. Finally, in vivo experiments in nude mice were implemented to assess the impact of exosome-delivered miR-15a on HCC. The exosomes from MSCs restrained HCC cell proliferative, migrating, and invasive potentials, and accelerated their apoptosis. miR-15a was expressed at low levels in HCC cells and could bind to SALL4, thus curtailing the proliferative, migrating, and invasive abilities of HCC cells. Exosomes successfully delivered miR-15a to HCC cells. Exosomal miR-15a depressed tumorigenicity and metastasis of HCC tumors in vivo. Overall, exosomal miR-15a from MSCs can downregulate SALL4 expression and thereby retard HCC development.

## Introduction

As a leading life-threatening malignancy, hepatocellular carcinoma (HCC) occupies ~85–90% of cases of primary liver cancers, which presents unsatisfying outcomes and limited therapeutic options [1, 2]. There exist factors for elevating the risk of HCC, like metabolic liver diseases, chronic infection of hepatitis B/C viruses, alcohol abuse, and consumption of foods contaminated with, for example, aflatoxins and aristolochic acid [3]. For patients with early-stage HCC, current managements, including tumor resection or ablation and liver transplantation, can achieve a clinical cure, while patients in advanced stages can only receive palliative treatments such as chemotherapy and pain relief [4]. Hence, there are still daunting challenges for improved HCC treatment calling for a better understanding of the molecular pathology.

Exosomes, as membranous vesicles, are responsible for transferring proteins, microRNAs (miRNAs or miRs), and long non-coding RNAs (lncRNAs) among cells, and represent promising new therapeutic targets for tumors [5, 6]. Mesenchymal stromal cells (MSCs) are frequently used as a source of cellular therapy because of their outstanding capacities for immunosuppression and regeneration [7]. Meanwhile, MSC-derived exosomes have been reported to be of great value in the experimental treatment of cancers [8]. For instance, recent research suggested that MSC-derived exosomes strongly inhibited HCC growth and progression [9]. miRNAs are involved in many biological pathways within a cell, and their dysregulation assumes a central role in cancer development [10]. For example, miR-15a is a suppressor for HCC progression by repressing cMyb, indicating it as a promising therapeutic target for HCC [11]. Moreover, exosomes secreted by miR-122-transfected adipose tissue-derived MSCs are capable of dramatically increasing the inhibiting efficacy of sorafenib on HCC progression by enhancing its chemosensitivity [12]. Bioinformatics analysis in a previous study identified spalt-like transcription factor 4 (SALL4) to be a putative target of miR-15b in hematopoietic stem cells [13]. SALL4 has been regarded as a crucial player in various types of cancers due to its ability to prevent the differentiation of stem cells and to enhance their self-renewal [14, 15]. Recent research investigating the interaction between miR-98 and SALL4 revealed that miR-98 plays a suppressive role in the development of HCC by inhibiting SALL4 directly [16]. Furthermore, the exosomal SALL4/miR-146a-5p axis has been revealed as a promising therapeutic and diagnostic target for applications in HCC [17]. Taken together, the above-mentioned findings suggest the influence of MSC-secreted exosomes in HCC, although the action of miR-15a enclosed by MSC-derived exosomes via SALL4 on HCC development remains enigmatic. Hence, we aimed in this study to further investigate the exosomal SALL4/miR-15a axis as a target for the clinical therapy of HCC.

## Results

### Successful characterization of MSCs and exosomes

**Fig. 1:**
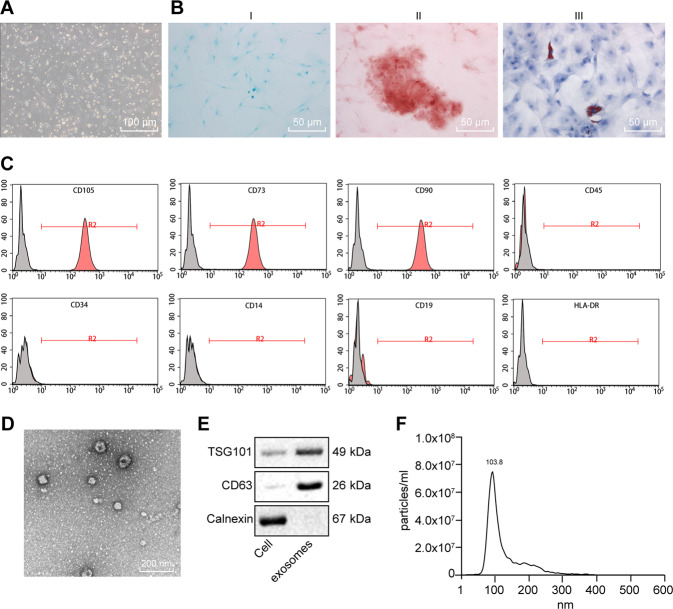
Identification of MSCs and exosomes. **A** Morphologic characteristics of MSCs at the fifth in culture were observed under an inverted microscope (×100). **B** Chondrocyte formation, osteoblast formation, and adipogenic differentiation were analyzed by cytochemical staining of Alcian blue (I, ×200), Alizarin Red, (II, ×200) and oil red O (III, ×200). **C** Expression of the surface marker proteins associated with MSCs was determined by flow cytometry. **D** Ultrastructure of exosomes was observed under a transmission electron microscope (×5000). **E** Representative western blots of the surface marker TSG101 and CD63 proteins. **F** Concentration and size of exosomes were detected by NTA analysis.

The morphology of isolated MSCs observed under a microscope revealed that MSCs grew in fibrous aggregates (Fig. 1A). Cytochemical staining analysis showed that MSCs could differentiate into osteoblasts, adipocytes, and chondrocytes in the corresponding media (Fig. 1B). As reflected by flow cytometry, putative MSCs positively expressed MSC surface markers (CD105, CD73, and CD90 proteins), but negatively expressed CD45, CD34, CD14, CD19, and HLA-DR (Fig. 1C), thus confirming their successful isolation.

The examination of exosomes from MSCs by ultracentrifugation, which revealed that isolated vesicles were circular and elliptical membranous shape with disk-like structures, and exhibited a complete envelope diameter of about 40–100 nm (Fig. 1D). We detected the expected surface marker proteins TSG101 and CD63, in the absence of the endoplasmic reticulum marker calnexin expression (Fig. 1E). Nanoparticle tracking analysis (NTA) analysis presented a mean particle size of 162.4 nm (ranging 50–200 nm) and that the particle size with the highest concentration was 133.8 nm at a concentration of 8 × 10^7^ particles/mL (Fig. 1F). The aforementioned results confirmed the successful isolation of exosomes.

### Exosomes from MSCs inhibit the proliferative, invasive, and migrating capacities of HCC cells in vitro

**Fig. 2:**
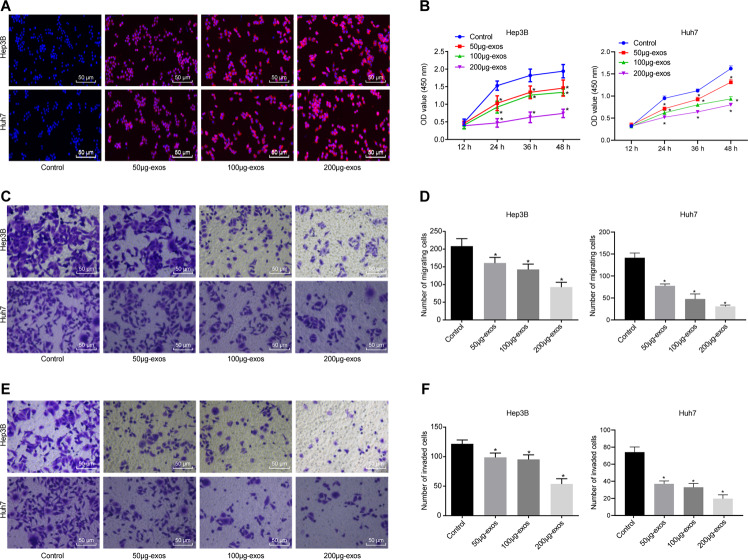
MSC-derived exosomes repress proliferation, migration, and invasion of HCC cells in vitro. **A** Uptake of MSC-derived exosomes from Hep3B and Huh7 cells was observed under a laser confocal microscope (×200). **B** Effect of exosomes on the proliferation of Hep3B and Huh7 cells was detected by CCK-8 assay. **C** Effect of exosomes on the migration of Hep3B cells and Huh7 was detected by transwell assay (×200). **D** Statistical analysis of panel **C**. **E** Effect of exosomes on the invasion of Hep3B and Huh7 cells was detected by transwell assay (×200). **F** Statistical analysis of panel E. *p* < 0.05 vs. control cells. Data in panel **B** were analyzed by repeated-measures ANOVA with Bonferroni post hoc testing, and in panels, **D** and **F** were analyzed by one-way ANOVA followed by Tukey’s post hoc test. Data are shown as mean ± standard deviation of three technical replicates.

Hep3B and Huh7 cells were co-cultured with 50, 100, and 200 μg PKH26-labeled exosomes for 24 h, respectively [18, 19]. Under a laser confocal microscope, the cells without exosome co-culture did not display any red fluorescence, while red fluorescence infiltration was observed in Hep3B and Huh7 cells co-cultured with exosomes in a concentration-dependent manner (Fig. 2A). Cell counting kit-8 (CCK-8) assay manifested lowered Hep3B and Huh7 cell viability when co-cultured with exosomes (Fig. 2B). Transwell assay indicated that the number of migrated cells and cells passing through the membrane was decreased in Hep3B and Huh7 cells co-cultured with exosomes, to a greater extent with increasing exosome concentration (Fig. 2C–F). Altogether, MSCs-secreted exosomes suppressed the proliferative, migrating, and invasive properties of HCC cells in a dose-dependent manner in vitro.

### miR-15a overexpression represses HCC cell proliferative, invasive, and migrating abilities in vitro

**Fig. 3:**
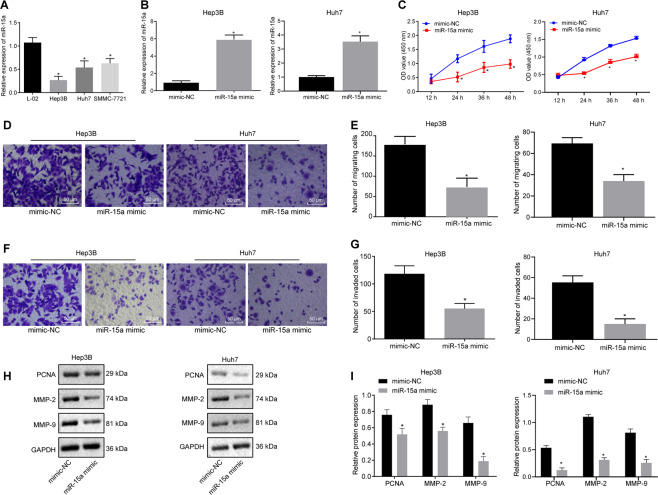
miR-15a suppresses the proliferation, migration, and invasion of HCC cells in vitro. **A** Expression of miR-15a was determined by RT-qPCR in normal hepatocytes L-02 and HCC cells Hep3B, Huh7, and SMMC-7721, relative to U6. **B** Expression of miR-15a was determined by RT-qPCR in Hep3B and Huh7 cells upon miR-15a mimic transfection, relative to U6. **C** Proliferation of Hep3B and Huh7 cells was detected by CCK-8 assay upon miR-15a mimic transfection. **D** Migration of Hep3B and Huh7 cells was detected by transwell assay upon miR-15a mimic transfection (×200). **E** Statistical analysis of panel **D**. **F** Invasion of Hep3B and Huh7 cells was detected by transwell assay upon miR-15a mimic transfection (×200). **G** Statistical analysis of panel **F**. **H** Representative western blots of MMP-2, MMP-9, and PCNA proteins in miR-15a mimic-transfected Hep3B and Huh7 cells. **I** The quantitation of panel **H**. *p < 0.05 vs. L-02 cells or Hep3B and Huh7 cells transfected with mimic-NC. Data in panel **C** were analyzed by repeated-measures ANOVA with Bonferroni post hoc testing, data in panels **B**, **E**, **G** and **I** were analyzed by unpaired *t*-test, and data in panel **A** were assessed by one-way ANOVA followed by Tukey’s post hoc test. Data are shown as mean ± standard deviation of three technical replicates.

Reverse transcription-quantitative polymerase chain reaction (RT-qPCR) suggested that miR-15a expression was decreased in all three HCC cell lines (Hep3B, Huh7, and SMMC-7721) compared with normal hepatocyte L-02, with the lowest expression in Hep3B and Huh7 cells. Hence, the following experimentation was implemented on these two cells lines (Fig. 3A). To investigate the impact of miR-15a on HCC cells, miR-15a expression was mimicked in Hep3B and Huh7 cells, in which RT-qPCR illustrated an increase in miR-15a expression (Fig. 3B). Moreover, miR-15a mimic decreased the viability (Fig. 3C) and the number of migrated cells and transmembrane cells (Fig. 3D–G) in Hep3B and Huh7 cells mimicking miR-15a expression. Western blot analysis presented a diminishment in matrix metallopeptidase (MMP)-2, MMP-9, and PCNA expression in the Hep3B and Huh7 cells mimicking miR-15a expression (Fig. 3H, I). Collectively, miR-15a elevation participated in repressing HCC cell proliferative, migrating, and invasive potentials in vitro.

### miR-15a overexpression suppresses proliferative, migrating, and invasive capacities of HCC cells by targeting SALL4 in vitro

**Fig. 4:**
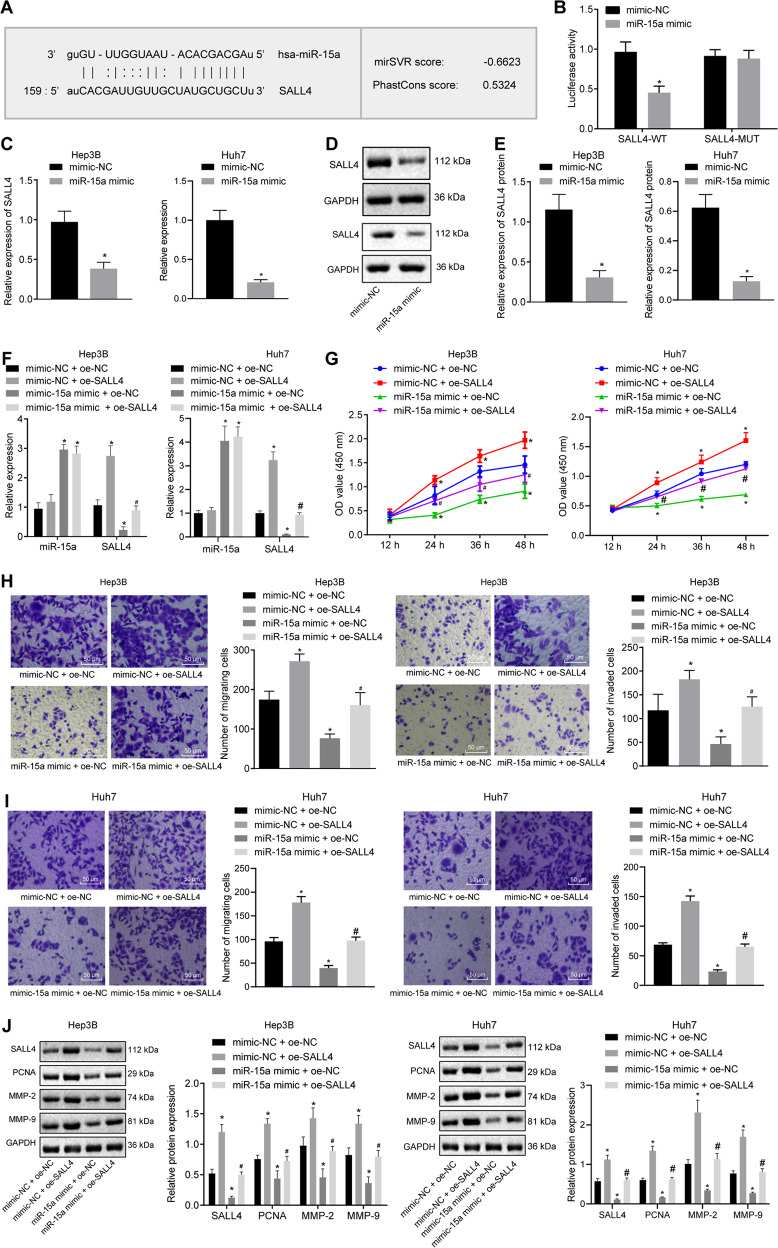
miR-15a reduces proliferation, migration, and invasion of HCC cells via SALL4 inhibition in vitro. **A** Putative miR-15a binding sites in the 3’UTR of SALL4 mRNA in the miRDB website (http://www.mirdb.org/). **B** miR-15a binding to SALL4 in cells verified by dual luciferase reporter gene assay. **C** mRNA expression of SALL4 was determined by RT-qPCR in miR-15a mimic-transfected Hep3B and Huh7 cells, relative to GAPDH. **D** Representative western blots of SALL4 protein in miR-15a mimic-transfected Hep3B and Huh7 cells, relative to GAPDH. **E** The quantitation of panel **D**. F Expression of miR-15a and SALL4 was determined by RT-qPCR in Hep3B and Huh7 cells transfected with miR-15a mimic and/or on-SALL4, relative to U6. **G** Proliferation of Hep3B and Huh7 cells were detected by CCK-8 following transfection with miR-15a mimic and/or oe-SALL4. **H** Migration of Hep3B and Huh7 cells was detected by transwell assay following transfection with miR-15a mimic and/or oe-SALL4 (×200). **I** Invasion of Hep3B and Huh7 cells was detected by transwell assay following transfection with miR-15a mimic and/or oe-SALL4 (×200). **J** Representative western blots of SALL4, MMP-2, MMP-9 and PCNA proteins in Hep3B and Huh7 cells following transfection with miR-15a mimic and/or oe-SALL4, relative to GAPDH. *p < 0.05 vs. Hep3B and Huh7 cells transfected with mimic-NC; #*p* < 0.05 vs. Hep3B and Huh7 cells transfected with miR-15a mimic+ of-NC. Data in panel **G** were analyzed by repeated-measures ANOVA with Bonferroni post hoc test. and data in panels **B**–**E** were analyzed by unpaired *t*-test. Data in panels **F**, **H**, **I**, and **J** were analyzed by one-way ANOVA followed by Tukey’s post hoc test. Data are shown as mean ± standard deviation of three technical replicates.

The miRDB website predicted that miR-15a could bind to the SALL4 gene (Fig. 4A). Dual-luciferase reporter gene assay displayed no changes in the luciferase activity of SALL4-mutation (MUT), while that of SALL4-wild type (WT) was diminished by miR-15a mimic (Fig. 4B). Moreover, RT-qPCR and Western blot assay demonstrated that SALL4 expression was decreased in the Hep3B and Huh7 cells in response to miR-15a mimic (Fig. 4C–E). Thus, SALL4 was a target gene of miR-15a in HCC cells.

To dissect out whether miR-15a can affect the development of HCC by regulating SALL4 gene expression, we stably transfected miR-15a mimic and/or overexpressed (oe)-SALL4 into the Hep3B and Huh7 cells. According to RT-qPCR results, SALL4 was up-regulated in Hep3B and Huh7 cells overexpressing SALL4, and miR-15a expression was elevated in Hep3B and Huh7 cells mimicking miR-15a expression. Furthermore, SALL4 expression was augmented in the cells following further transfection of of-SALL4 in the presence of miR-15a mimic (Fig. 4F).

CCK-8 (Fig. 4G) and Transwell (Fig. 4H, I) assays demonstrated that the viability and the number of migrated cells and transmembrane cells of Hep3B and Huh7 cells after SALL4 overexpression tended to increase, while miR-15a mimic induced opposite effects. Moreover, the viability and the number of migrated cells and transmembrane cells of Hep3B and Huh7 cells following treatment with miR-15a mimic and oe-SALL4 were higher than miR-15a mimic treatment alone.

As documented by Western blot analysis, SALL4, MMP-2, MMP-9, and PCNA expression was augmented in Hep3B and Huh7 cells overexpressing SALL4 but reduced in miR-15a mimic-transfected cells, which was nullified upon dual treatment with miR-15a mimic and oe-SALL4 (Fig. 4J). To sum up, SALL4 can reverse the inhibiting effects of miR-15a on the proliferative, migrating, and invasive properties of HCC cells in vitro.

### Exosomes derived from MSCs deliver miR-15a to HCC cells to repress HCC cell proliferative, migrating, and invasive potentials but to promote apoptosis in vitro

**Fig. 5:**
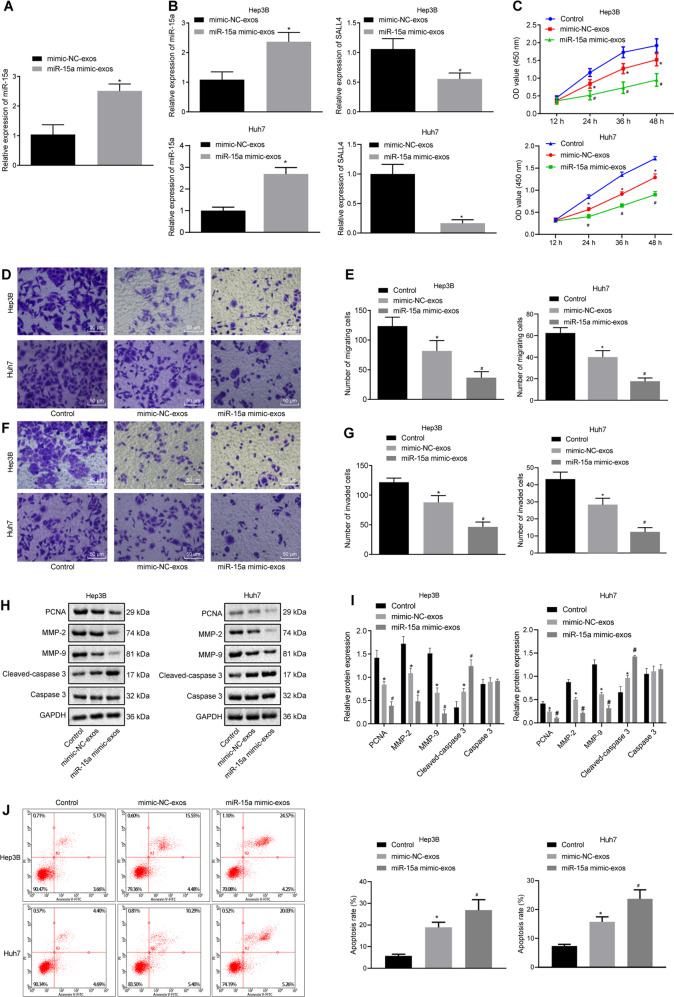
Exosomes transfer miR-15a to HCC cells and thus affect proliferation, migration, invasion, and apoptosis of HCC cells. **A** Expression of miR-15a was determined by RT-qPCR in exosomes from the miR-15a mimic-treated MSCs, relative to U6 (*p < 0.05 vs. exosomes from the MSCs transfected with mimic-NC). **B** Expression of miR-15a and SALL4 was determined by RT-qPCR in the co-culture system of Hep3B and Huh7 cells and miR-15a mimic-exos, relative to U6 and GAPDH, respectively. **C** Hep3B and Huh7 cell proliferation was detected by CCK-8 following co-culture with miR-15a mimic-exos. **D** Hep3B, and Huh7 cell migration were detected by Transwell assay following co-culture with miR-15a mimic-exos (×200). **E** Statistical analysis of panel **D**. **F** Hep3B and Huh7 cell invasion was detected by Transwell assay following co-culture with miR-15a mimic-exos (×200). **G** Statistical analysis of panel **F**. **H** Representative western blots of Cle-caspase3, caspase3, MMP-2, MMP-9, and PCNA proteins in the co-culture system of Hep3B and Huh7 cells and miR-15a mimic-exos, relative to GAPDH. **I** The quantitation of panel **H**. **J** The apoptosis of Hep3B and Huh7 cells detected using flow cytometry. *p < 0.05 vs. control Hep3B and Huh7 cells; #*p* < 0.05 vs. Hep3B and Huh7 cells co-cultured with mimic-NC-exos. Data in panel **C** were analyzed by repeated-measures ANOVA with Bonferroni post hoc test, and data in panels **A** and **B** were analyzed by unpaired *t*-test. Data in panels **E**, **G**, **I**, and **J** were analyzed by one-way ANOVA, with Tukey’s post-hoc test. Data are shown as mean ± standard deviation of three technical replicates.

To obtain exosomes with high expression of miR-15a, exosomes were isolated from the bone marrow MSCs following transfection. miR-15a expression was enhanced in the isolated exosomes from MSCs mimicking miR-15a expression (Fig. 5A). To investigate whether exosomes can deliver miR-15a to affect the pathogenesis of HCC, miR-15a mimic-exos were co-cultured with Hep3B and Huh7 cells. The results presented miR-15a expression was augmented while SALL4 expression was restrained in the co-culture system of Hep3B and Huh7 cells and miR-15a mimic-exosomes (exos) (Fig. 5B). This result confirms the ability of exosomes to deliver miR-15a to Hep3B and Huh7 cells, thereby inhibiting the expression of SALL4. Moreover, Hep3B and Huh7 cell viability (Fig. 5C) and the number of migrated and invasive Hep3B and Huh7 cells (Fig. 5D–G) were reduced upon co-culture with exosomes, especially upon co-culture with miR-15a mimic-exos. Western blot analysis manifested a reduction in protein expression of MMP-2, MMP-9, and PCNA but elevation in Cle-caspase3/caspase3 expression in the co-culture system of Hep3B and Huh7 cells with exosomes, especially after co-culture with miR-15a mimic-exos (Fig. 5H, I). In addition, flow cytometry revealed that the apoptosis of Hep3B and Huh7 cells were higher upon treatment with mimic-NC-exos, while Hep3B and Huh7 cell apoptosis was facilitated following co-culture with miR-15a mimic-exos compared with co-culture with mimic-NC-exos (Fig. 5J). Taken together, exosomes derived from bone marrow MSCs could transmit miR-15a to HCC cells, thus inhibiting HCC cell proliferative, migrating, and invasive abilities but enhancing apoptosis in vitro.

### Exosomes transfer miR-15a to delay the growth of HCC tumors in vivo

**Fig. 6:**
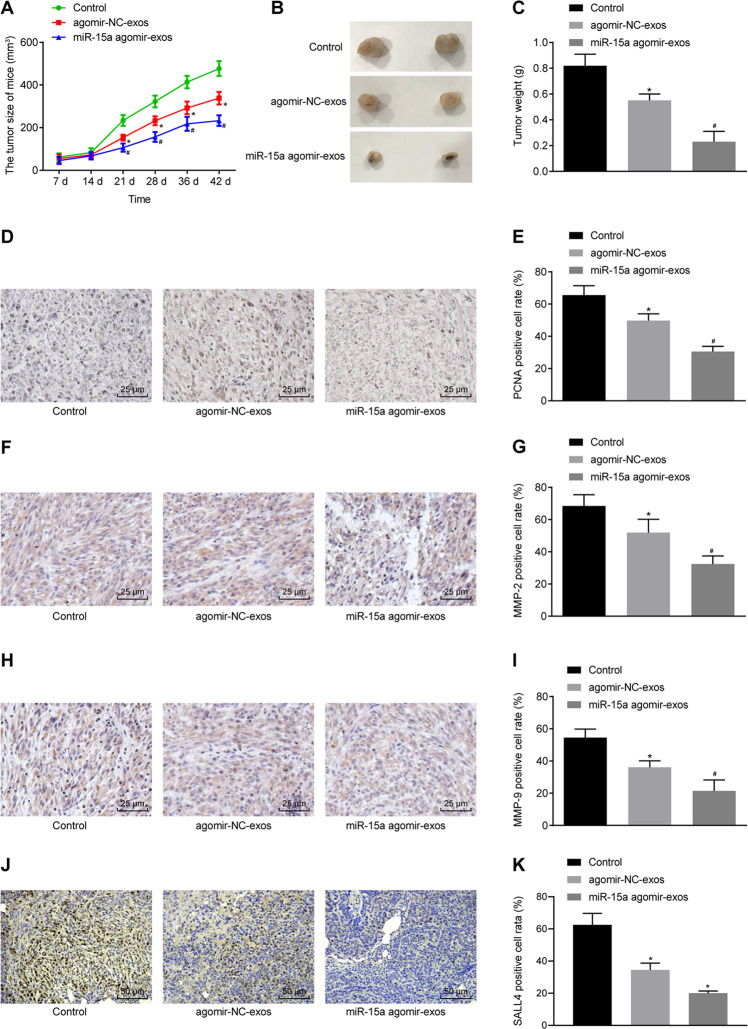
Delivery of miR-15a by exosomes prevents tumor growth in vivo. **A** The growth of the HCC xenograft tumor was measured every 7 days in nude mice injected with Hep3B cells treated with miR-15a agomir-exos. **B** Macroscopic observation of xenograft tumor of nude mice injected with Hep3B cells treated with miR-15a agomir-exos. **C** Tumor weight of nude mice injected with Hep3B cells treated with miR-15a agomir-exos. **D** Positive expression rate of PCNA protein was examined by immunohistochemistry in tumor tissues of nude mice injected with Hep3B cells treated with miR-15a agomir-exos (×400). **E** The quantitation of panel **D**. **F** Positive expression rate of MMP-2 protein was examined by immunohistochemistry in tumor tissues of nude mice injected with Hep3B cells treated with miR-15a agomir-exos (×400). **G** The quantitation of panel **F**. **H** Positive expression rate of MMP-9 protein was examined by immunohistochemistry in tumor tissues of nude mice injected with Hep3B cells treated with miR-15a agomir-exos (×400). **I** The quantitation of panel **H**. **J** Positive expression rate of SALL4 protein was examined by immunohistochemistry in tumor tissues of nude mice injected with Hep3B cells treated with miR-15a agomir-exos (×400). **K** The quantitation of panel **J**. *p < 0.05 vs. control mice; #*p* < 0.05 vs. mice injected with agomir-NC-exos-treated Hep3B cells. Data in panel **A** were analyzed by repeated-measures ANOVA with Bonferroni test, and data in panels **C**, **E**, **G**, **I**, and **K** were analyzed by one-way ANOVA, with Tukey’s post-hoc test. *n* = 12 for mice following each treatment.

To ascertain whether the delivery of miR-15a by exosomes affects the tumorigenicity of HCC in vivo, nude mice were subcutaneously inoculated with Hep3B cells treated with miR-15a agomir-exos. Tumor volume and weight were reduced in the mice treated with exosomes, and these trends were further promoted in the mice treated with miR-15a agomir-exos (Fig. 6A–C).

As exhibited by immunohistochemistry, the positive rates of SALL4, MMP-2, MMP-9, and PCNA proteins were decreased in tumor tissues of mice treated with exosomes, and these trends were further facilitated in the tumor tissues of mice treated with miR-15a agomir-exos (Fig. 6D–K). Conclusively, delivery of miR-15a by exosomes suppressed the growth of HCC tumor in vivo.

## Discussion

HCC accounts for ~90% of all newly diagnosed cases of primary liver cancer, which represents a burgeoning cause of cancer-related mortality worldwide [20]. Currently, the main treatments of HCC are resection or liver transplantation, but improved molecular therapies, are widely used to augment the survival of HCC patients [21]. Exosomes, which are implicated in the formation and mediation of tumor stroma by MSCs, have been found to modulate communication among cells through transferring genetic information to receiver cells via their cargos of coding and non-coding RNAs [22]. However, the study regarding MSC-derived exosomes in HCC and its downstream mechanism remains at an early stage. Therefore, we investigated the molecular mechanism of MSC-derived exosomes in HCC and discovered that delivery of miR-15a by MSC-derived exosomes delayed the development of HCC by targeting SALL4.

The main finding of this study was that MSC-derived exosomes could depress the proliferative, invasive, and migrating capacities of HCC cells. Previously, exosomes have been implicated in inhibiting HCC cell proliferative and invasive potentials in vitro and in vivo [23]. Besides, MSC-derived exosomes can pass into a solid tumor site and transfer the repressive oligonucleotides into the tumor tissues, with suppressive effects on the progression of breast cancer [24]. We next detected the effect of miR-15a on HCC cells and found decreased expression of MMP-2, MMP-9, and PCNA in HCC cells transfected with miR-15a mimic, indicating that miR-15a elevation also plays an inhibiting role in the proliferation, migration, and invasion of HCC cells. Consistent with our present findings, another study also identified miR-15a as a potential therapeutic target for HCC due to its suppressive functions on the migration ability of HCC cells [11]. Also, miR-15a was investigated as a crucial mediator in weakening the proliferation ability of colorectal cancer cells and laryngeal cancer cells, with the ability to inhibit the progression of these two cancers [25, 26]. In addition, matrix metalloproteinases (MMPs) are important participants of many pathological processes in relation to cancer [27]. Down-regulation of MMP-2 and MMP-9 by diosmetin can suppress the biological characteristics of HCC cells [28]. PCNA, known as a key mediator of cell cycle and apoptosis, is involved in the development of cancers such as non-small cell lung cancer and gastric cancer, in which overexpressed PCNA acts as an oncogene [29–31]. There is also a reduction of PCNA expression in association with reduced HCC tumor formation [32]. To sum up, the present and earlier findings concur in validating the suppressing capacity of MSC-derived exosomes and miR-15a on HCC.

Another key observation of the current study was that miR-15a could be transferred into HCC cells by MSC-derived exosomes and then inhibited their proliferation, migration, and invasion by reducing the expression of SALL4. Similarly, previous research showed that miR-122 could be packaged into exosomes derived from MSCs, and dramatically enhanced the antitumor function of sorafenib on HCC [12]. SALL4 is overexpressed in diseases such as cervical cancer and lung cancer and is associated with enhanced tumorigenesis and tumor progression [33, 34]. Furthermore, the expression of SALL4 was found to be reduced by miR-15b, thus contributing to the suppressed proliferation, migration, and invasion of glioma cells [35]. Besides, the expression of SALL4 can also be suppressed by miR-98, thus presenting the miR-98/SALL4 axis as a promising candidate for HCC treatment [16]. Furthermore, SALL4 can interact with exosomal miR-146a-5p to modulate the progression of HCC [17]. Taken together, miR-15a transferred by MSC-derived exosomes may have the capacity to regulate the progression of HCC by expects on SALL4.

**Fig. 7:**
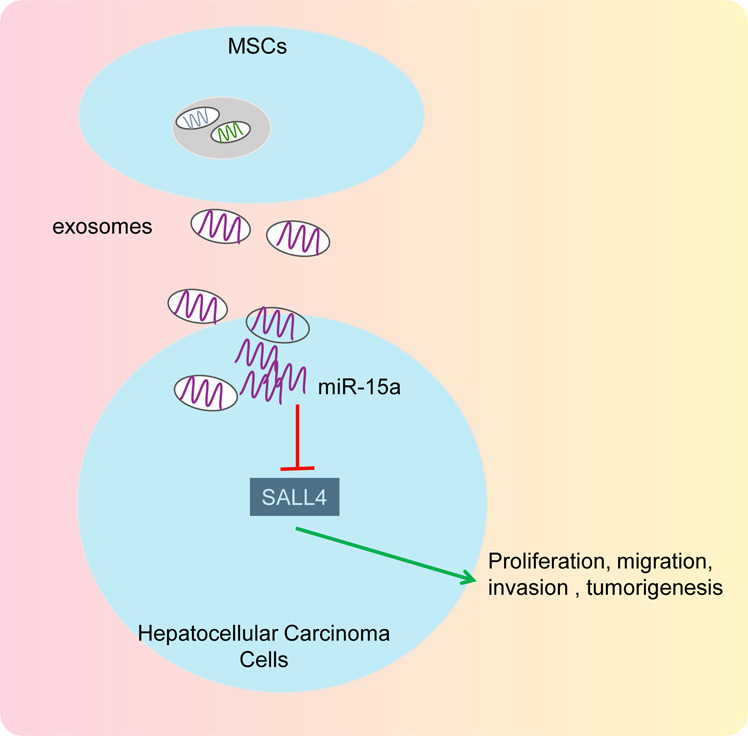
A schematic for the mechanism and function of MSC-derived exosomal miR-15a in HCC via SALL4. MSC-derived exosomes were capable of transmitting miR-15a, which thus inhibited the expression of SALL4, and thereby inhibiting the proliferation, migration, and invasion of HCC cells in vitro as well as retarding the tumorigenesis in vivo, ultimately preventing the occurrence of HCC.

In summary, this study provides new evidence demonstrating that MSC-derived exosomes can transfer miR-15a to HCC cells to inhibit the proliferation, migration, and invasion by negatively regulating SALL4 (Fig. 7). Hence, MSC-derived exosomes may represent a promising new direction for HCC treatment. However, further studies were needed to support translational clinical studies of MSC-derived exosomes on HCC management.

## Materials and methods

### Ethics statement

This study was approved and guaranteed by Shanghai Tenth People’s Hospital, Tongji University School of Medicine (Approval No. SHSY-IEC-15-19). Written informed consent was offered by each participant. Animal experiments were implemented as per the recommendations in the Guide for the Care and Use of Laboratory Animals outlined by the US National Institutes of Health. All efforts were made to minimize the number and discomfort of the included animals.

### Isolation and identification of MSCs

Bone marrow samples were attained from three in-patients (two males and one female, aged 26–52 years) diagnosed with osteonecrosis of the femoral head (ONFH) at the Shanghai Tenth People’s Hospital, Tongji University School of Medicine, Jiangxi Provincial Tumor Hospital, and Cancer Institute, Nantong Tumor Hospital. They were diagnosed as per magnetic resonance imaging results. The patients without loss of femoral head and without diseases like trauma, blood system, tuberculosis, and tumor infiltration were selected. MSCs were isolated from three donated samples in the light of a published method [36] and incubated in Dulbecco’s modified Eagle’s medium/Ham’s F-12 medium (DMEM-F12) (Hyclone, South Logan, UT, USA) encompassing 10% fetal bovine serum (FBS; 10099141, GIBCO BRL, Grand Island, NY, USA) and 0.2% penicillin and streptomycin (Hyclone). MSCs were subcultured every 3 days, and the following experimentation was implemented with MSCs at passages 3–7. According to manuals for the incubation media, MSCs were incubated in OriCell™ MSCs osteogenesis, adipogenesis, and cartilage differentiation media (Cyagen, Guangzhou, Guangdong, China), respectively, and their differentiation was confirmed using Alizarin red staining, oil red O staining, and alcian blue staining, respectively.

After three times of subculture, MSCs were digested, centrifuged and incubated with mouse monoclonal antibodies (Abcam, Cambridge, UK) to CD105 (ab11414, 1:100), CD73 (ab239246, 1:50), CD90 (ab226, 1:100), CD45 (ab27287, 1:50), CD34 (ab131589, 1:50), CD14 (ab28061, 1:200), CD19 (ab24936, 1:50), or HLA-DR (ab1182, 1:50). Additionally, the same concentration of goat anti-mouse isotope-marked antibodies against immunoglobulin G (IgG; BD Biosciences Pharmingen, San Jose, CA, USA, 1:1000) conjugated with fluorescein isothiocyanate (FITC) were taken as negative control (NC). Samples were detected by FACSVerse (BD Biosciences Pharmingen), followed by result analysis using FlowJo software (Tree Star, Ashland, OR, USA).

### Cell culture

Incubation of human HCC cell lines Hep3B and Huh7 (the Cell Bank of the Chinese Academy of Sciences, Shanghai, China) was implemented in DMEM (GIBCO BRL) encompassing 10% FBS, 100 units/mL penicillin, and 100 g/mL streptomycin. Normal hepatocytes L-02 and human HCC cell line SMMC-7721 were acquired from American Type Culture Collection (Manassas, VA, USA) and incubated in Roswell Park Memorial Institute (RPMI) 1640 medium (HyClone) encompassing 15% FBS and 0.2% penicillin–streptomycin. These cells were cultured in a 5% CO_2_ at 37 °C incubator (Thromo3111, Beisheng medical device Co., Ltd, Jinan, Shandong, China) and subcultured every 3 days.

### Exosome isolation

The exosomes in the serum was discarded by ultra-centrifuging the culture medium encompassing serum at 100,000×*g* and 4 °C. MSC incubation was conducted in the conditioned medium (DMEM-F12 and 10% serum without exosomes) for 48 h. Logarithmically growing MSCs were cultured and the supernatant was collected to extract exosomes. Subsequent to the removal of cells by 10-min MSC centrifugation at 500×*g* and 4 °C, the supernatant was re-centrifuged for 20 min at 10,000×*g* and 4 °C to discard extracellular vesicles. A 0.22-μm filter was adopted to filter cells before 70-min cell centrifugation at 110,000×*g* and 4 °C. After re-suspending in PBS, the pellet was centrifuged again as before. Finally, cells were resuspended in 100 μL sterile PBS and then frozen at −20 °C for the following experimentation.

### Transmission electron microscopy (TEM)

Subsequent to ultra-centrifugation of the exosome resuspension solution, the pellet was fixed with 2% paraformaldehyde and 2.5% glutaraldehyde for 1 h at 4 °C and then in 1% tannin for 1.5 h. Then pellets underwent gradient alcohol dehydration, permeating, overnight embedding, and 24-h polymerization at 35, 45, and 60 °C. The pellets underwent ultrathin sectioning and were stained with lead uranium before TEM observation.

### NTA

The resuspended exosome solution was diluted and supplemented with a nanoparticle tracking analyzer (Malvern Panalytical, Worcestershire, UK). Subsequent to attaining the diluted samples with a concentration fluctuation of (1–9)×10^8^ cells/mL, we selected an appropriate background gray scale and recorded their movement trajectory. We then calculated the concentration of diluted samples and the distribution of their particle size to assess the exosome concentration of the stock solution.

### Western blot analysis

Total protein was isolated from MSCs using radioimmunoprecipitation assay lysis buffer (R0010, Solarbio, Beijing, China) encompassing phenylmethylsulfonyl. Following dissolving in 2× sodium dodecyl sulfate (SDS) loading buffer, 50 μg protein received 5-min boiling at 100 °C. These samples were separated using 10% SDS–polyacrylamide gel electrophoresis and electroblotted to polyvinylidene fluoride membranes. Next, the membranes were incubated with diluted rabbit monoclonal antibody (Abcam) to TSG101 (ab30871, 1:2000), and rabbit polyclonal antibodies (Abcam) to CD63 (ab118307, 1:50), calnexin (ab75801, 1:2000), SALL4 (ab31968, 1:50), MMP-2 (ab37150, 1:100), MMP-9 (ab73734, 1:100), Cle-caspase-3 (ab2302, 1:1000), caspase-3 (ab4051, 1:1000), and glyceraldehyde-3-phosphate dehydrogenase (GAPDH, ab8245, 1:10,000) overnight at 4 °C. Subsequently, the membranes underwent 1-h incubation with horseradish peroxidase-tagged secondary antibody to IgG (1:1000). The immunocomplexes on the membrane received visualization with enhanced chemiluminescence reagent (BB-3501, Amersham, Little Chalfont, UK) before quantification of the band intensities with a IS gel image analysis system and an Image J software.

### Exosome uptake assay

Exosomes were labeled with pyridoxal kinase 26 (PKH26) fluorescent dye (Sigma-Aldrich, St. Louis, MO, USA): 4 μL of PKH26 fluorescent dye was supplemented to 1 mL Dilument C (Beyotime, Shanghai, China) solution to prepare 4 µM PKH26 dye, which was then added with 200 μg of exosomes. The above two solutions were subsequently mixed gently for five min and then centrifuged for 2 h at 100,000×*g* and 4 °C for 2 h, resuspended, and centrifuged again as before prior to the collection of the fluorescent-labeled exosomes.

### Cell and exosome transfection

HCC cells were seeded into a six-well plate encompassing DMEM at 24 h before transfection. The confluent 70% was resuspended in DMEM without serum and seeded into another six-well plate. Thereafter, the cells were transfected with mimic-NC, miR-15a mimic, or-NC, and oe-SALL4 plasmids. Subsequent to overnight transfection, the medium was renewed with DMEM encompassing 10% exosome-depleted FBS (EXO-FBS-50A-1, System Biosciences, Palo Alto, CA, USA) and 1% penicillin–streptomycin (Tianhang Biotechnology, Hangzhou, China) [37] for subsequent experiments. All these plasmids were acquired from RiboBio Company (Guangdong, China). MSC transfection was implemented with mimic-NC and miR-15a mimic plasmids using the same method. Following 24-h transfection, mimic-NC-exos and miR-15a mimic-exos were extracted from MSCs.

### RNA isolation and quantitation

Total RNA was isolated from cells or exosomes using TRIzol reagents (16096020, Thermo Fisher Scientific, Waltham, MA, USA). Then, according to the instructions of a cDNA kit (K1622; Fermentas Inc., Ontario, CA, USA), 5 µg RNA was reversely transcribed into complementary DNA (cDNA), and RNA of miRNA was reversely transcribed into cDNA using miRNA first-strand cDNA synthesis (tailing method) kit (Sangon Biotech, Shanghai, China; B532451-0010), which was diluted into 50 ng/µL and subjected to PCR amplification. During amplification, 25 µL total reaction system was applied, which included 300 ng cDNA (for gene expression detection) or 100 ng cDNA (for miRNA expression analysis) as templet, 1× PCR buffer, 200 μmol/L dNTPs, 80 pmol/L forward primers, 80 pmol/L reverse primers and 0.5 U Taq enzyme (S10118, Shanghai yuanye Bio-Technology Co., Ltd., Shanghai, China). The reaction conditions included 5-min predenaturation at 94 °C, 30 cycles of 30-s denaturation at 94 °C, 30-s annealing at 54.5 °C and 30-s extension at 72 °C, and 10-min extension at 72 °C. miR-15a expression was normalized to U6 (their reverse primers were provided directly by Sangon). SALL4 expression was determined using PrimeScript RT-PCR kits (TaKaRa, Tokyo, Japan) standardized by GAPDH. The primer sequences are presented in Supplementary Table 1. The 2^−ΔΔCt^ method was applied for the calculation of the fold changes.

### Dual-luciferase reporter gene assay

Synthetic SALL4 3′ untranslated region (UTR) gene segment was introduced into pMIR-reporter (Beijing Huayueyang Biotechnology Co., Ltd., Beijing, China) by endonuclease site SpeI and Hind III. The complementary sequence MUT site of the seed sequence was designed on SALL4 WT, and the target fragment was inserted into the reporter plasmid of pMIR-reporter by digestion of restriction endonuclease using T4 DNA ligase. The correctly sequenced WT and MUT luciferase reporter plasmids were co-transfected with miR-15a mimic into HEK-293T cells (CRL-1415, Xinyu Biotechnology Co., Ltd., Shanghai, China). Subsequent to 48-h transfection, the cells were lysed, after which the luciferase activity was detected using a Glomax 20/20 luminometer fluorescence detector (Promega, Madison, WI, USA) and a luciferase assay kit (RG005, Beyotime).

### CCK-8 assay

Cells that were co-cultured with exosomes were incubated in a 96-well plate at 1.5 × 10^3^ cells/well with three replicates set for each well. Cells were incubated with CCK-8 solution for 2 h at the end of 12, 24, 36, and 48 h of culture, and the optical density value *A* was assessed at 450 nm with an enzyme-linked immunosorbent detector.

### Transwell assay

Cell migration measurement was conducted. In short, logarithmically growing MSCs were incubated in a six-well plate before 48-h co-incubation with exosomes. The cells were then treated with 200 μL of 0.25% trypsin and resuspended with DMEM without serum to a density of 3 × 10^5^ cells/mL. Then, 100 μL cell suspension was supplemented into the apical chamber of a transwell chamber.

Next, cell invasion was determined. In brief, the apical chamber coated with 50 μL matrigel was supplemented with cell suspension, and the basolateral chamber was supplemented with 500 μL DMEM encompassing 10% FBS. Then, the transwell chamber was placed in a 5% CO_2_ and 37 °C incubators for 24 h, followed by formaldehyde fixation (10 min) and crystal violet staining (10 min). The upper cells of the chamber were carefully wiped with a cotton swab, and the cell membrane was removed and sealed with neutral resin in a slide. Six random fields were examined under a microscope for counting.

### Flow cytometry

The cells underwent 10-min staining with Annexin V-FITC and propidium iodide (PI) in the apoptosis kit (APOAF-20TST, Sigma) at 4 °C. The apoptosis rate (1 × 10^5^ cells/sample) was detected on the CytoFlex flow cytometer (Beckman Coulter Inc., Brea, CA, USA), followed by data analysis with a FlowJo software (version 7.0; FlowJo LLC)

### Xenograft tumor in nude mice

A total of 36 specific pathogen-free (SPF) BALB/c nude mice (aged 3–4 weeks; Shanghai SLAC Laboratory Animal Co., Ltd., Shanghai, China) were conventionally raised in an SPF environment. HCC cells (1.5 × 10^6^) were subjected to different treatments: HCC cells were cultured in serum-free medium, in serum-free medium encompassing 200 μg agomir-NC-exos, or in serum-free medium encompassing 200 μg of miR-15a agomir-exos, respectively. Following 12 h, the exosomes remaining outside HCC cells were washed away thoroughly with PBS, and groups of 12 nude mice were injected subcutaneously with the above HCC cells. The tumor length (*L*) and width (*W*) of nude mice with different treatments were measured with digital calipers on days 7, 14, 21, 28, 36, and 42 after inoculation. The volume (*V*) of tumors was calculated using the formula: *V* = *L* × *W*^2^ × 0.5 [38], and a growth curve was plotted. All nude mice were euthanatized 6 weeks after inoculation, whereupon the tumors were excised and weighed, followed by immersion fixation in 4% formaldehyde solution for immunohistochemical examination.

### Immunofluorescence assay

Fresh tumor tissues obtained from nude mice were sliced into 4 μm subsequent to 10% neutral formalin fixing, gradient alcohol dehydration, xylene clearing, and paraffin embedding. The slices were dewaxed and hydrated with conventional gradient alcohol before blocking of endogenous peroxidase activity with distilled water encompassing 0.3% hydrogen peroxide. The slices were subsequently blocked with FBS for 15 min. Next, the slices were probed overnight at 4 °C with primary rabbit polyclonal antibodies (Abcam) to MMP-2 (ab97779, 1:500), MMP-9 (ab38898, 1:1000), proliferating cell nuclear antigen (PCNA, ab18197, 1:100), and SALL4 (ab29112, 1:1000). The slices underwent 30-min re-probing with goat anti-rabbit IgG (ab150077, 1:500, Abcam) warmed in a water bath at 37 °C before 2-min hematoxylin counterstaining, dehydration, clearing, and sealing. The slices were observed under an inverted microscope and photographed. PBS was taken as a NC instead of primary antibodies.

### Statistical analysis

All data were processed with the SPSS 21.0 statistical software (IBM Corp., Armonk, NY, USA). The measurement data obeying normal distribution and homogeneity of variance were expressed as mean ± standard deviation. Comparisons between two groups were analyzed using unpaired *t*-test, while comparisons among multiple groups were assessed by one-way analysis of variance (ANOVA) followed by Tukey’s post hoc test. Data comparisons at different time points were analyzed using repeated-measures ANOVA followed by Bonferroni’s test. A value of *p* < 0.05 was considered to be statistically significant.

## Supplementary information

Authorshipform_springer_nature

cddiscovery-author-contribution-form

Table S1
