# Supplementary figures and images for: Exosomal microRNA-15a from mesenchymal stem cells impedes hepatocellular carcinoma progression via downregulation of SALL4
Source: Cell Death Discov. 2021 Aug 28;7:224. doi: 10.1038/s41420-021-00611-z (PMC8403170; doi:10.1038/s41420-021-00611-z)

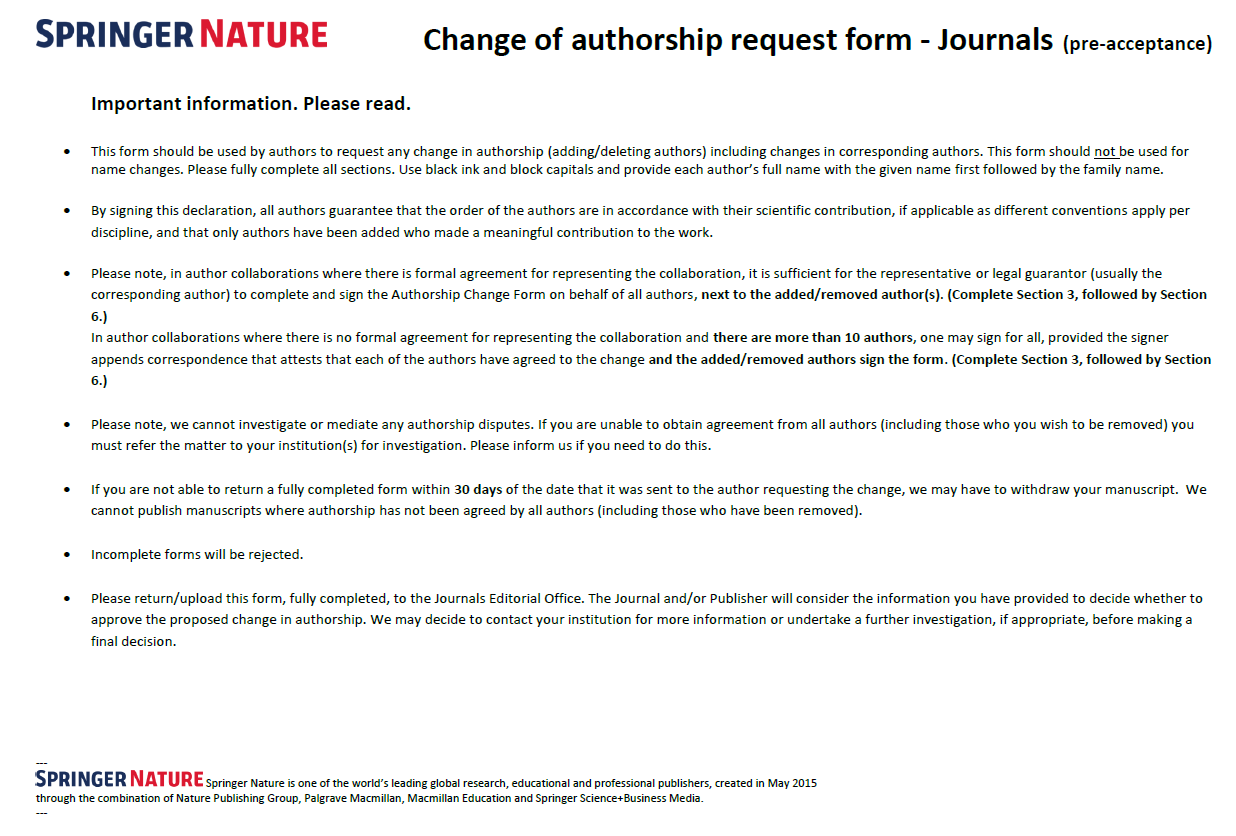


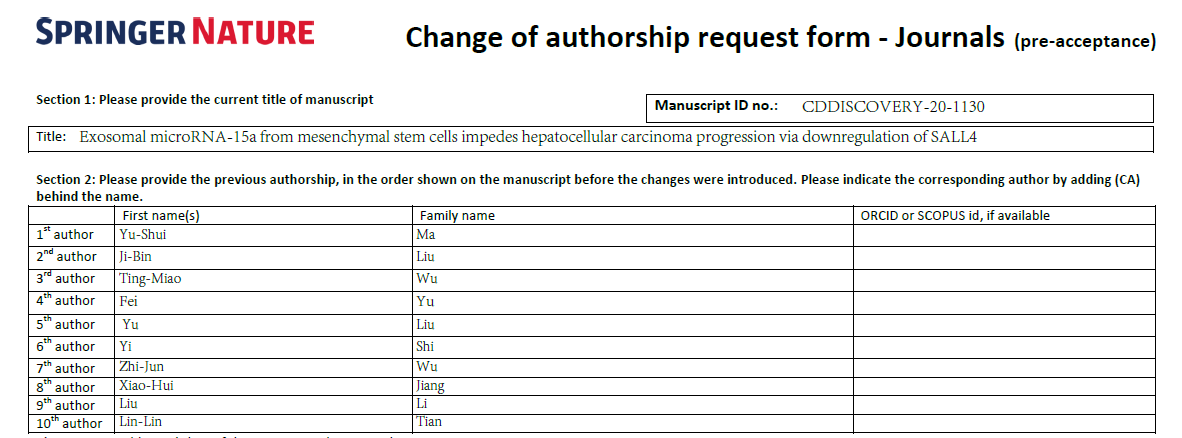


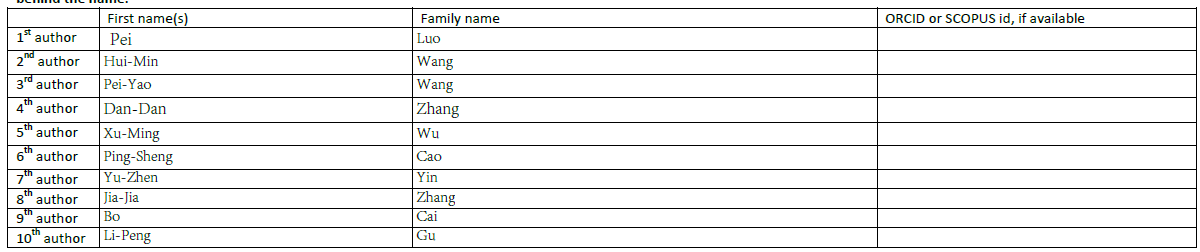


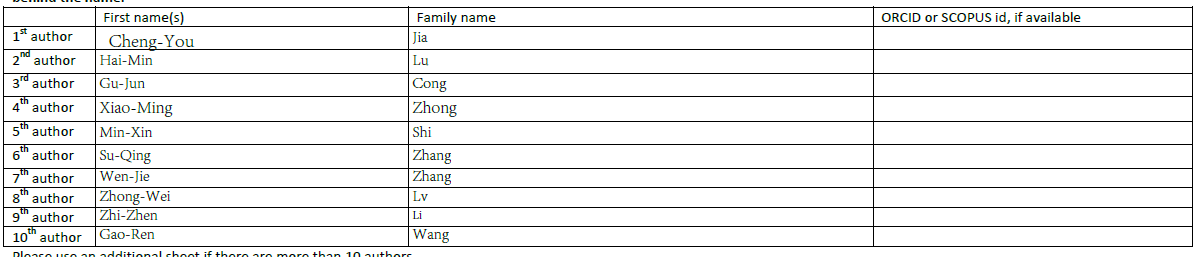


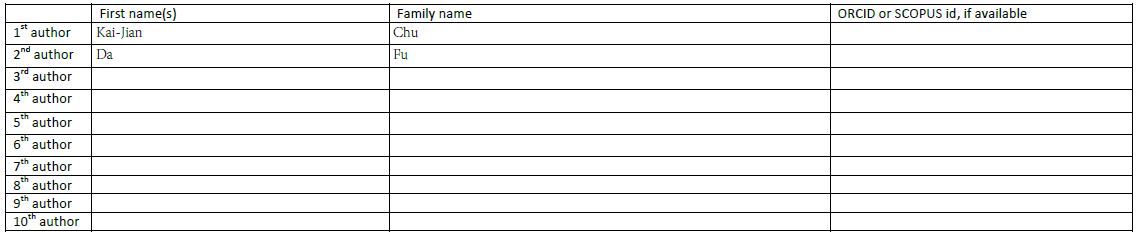


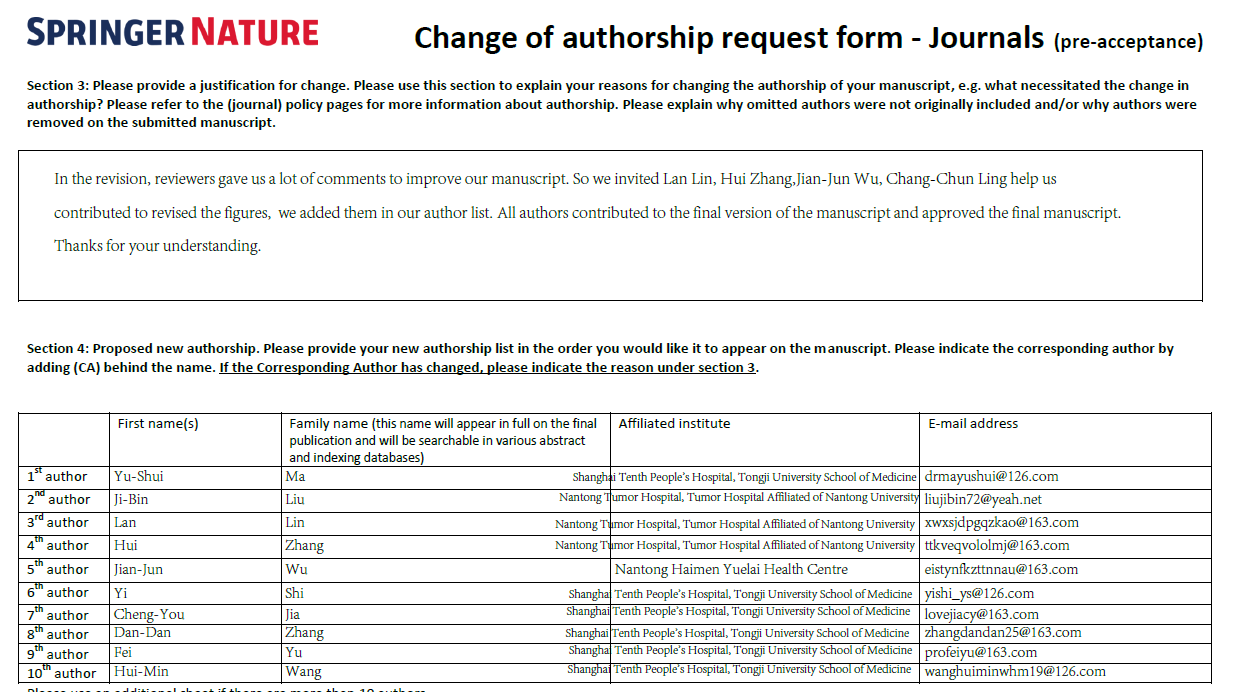


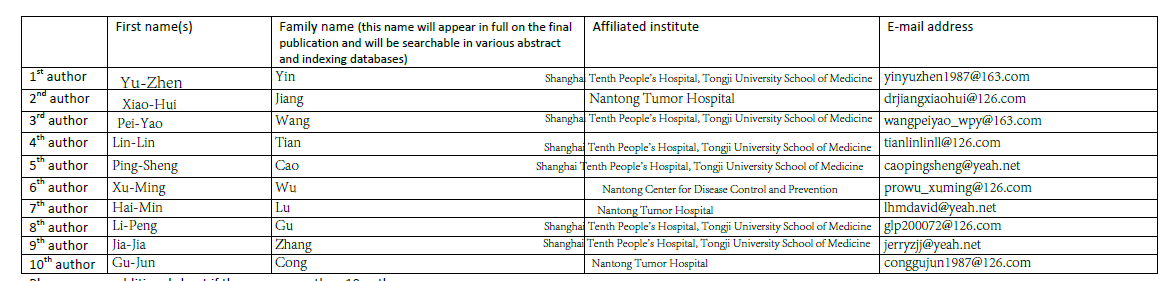


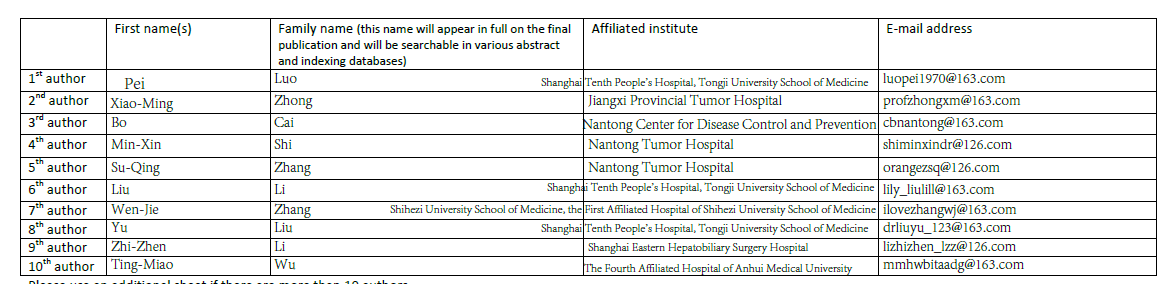


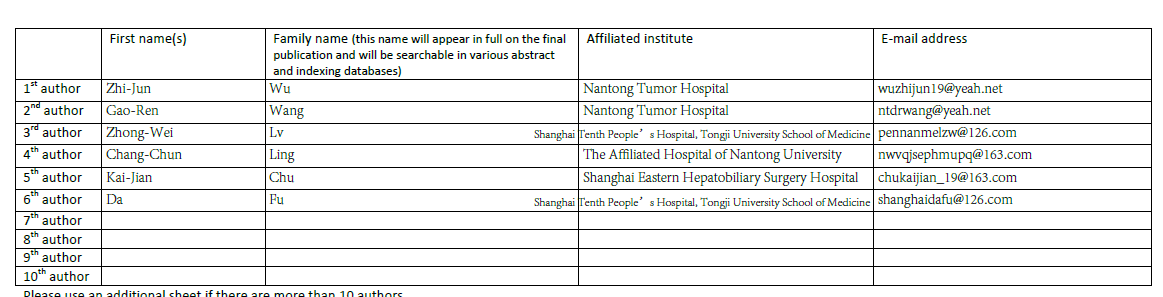


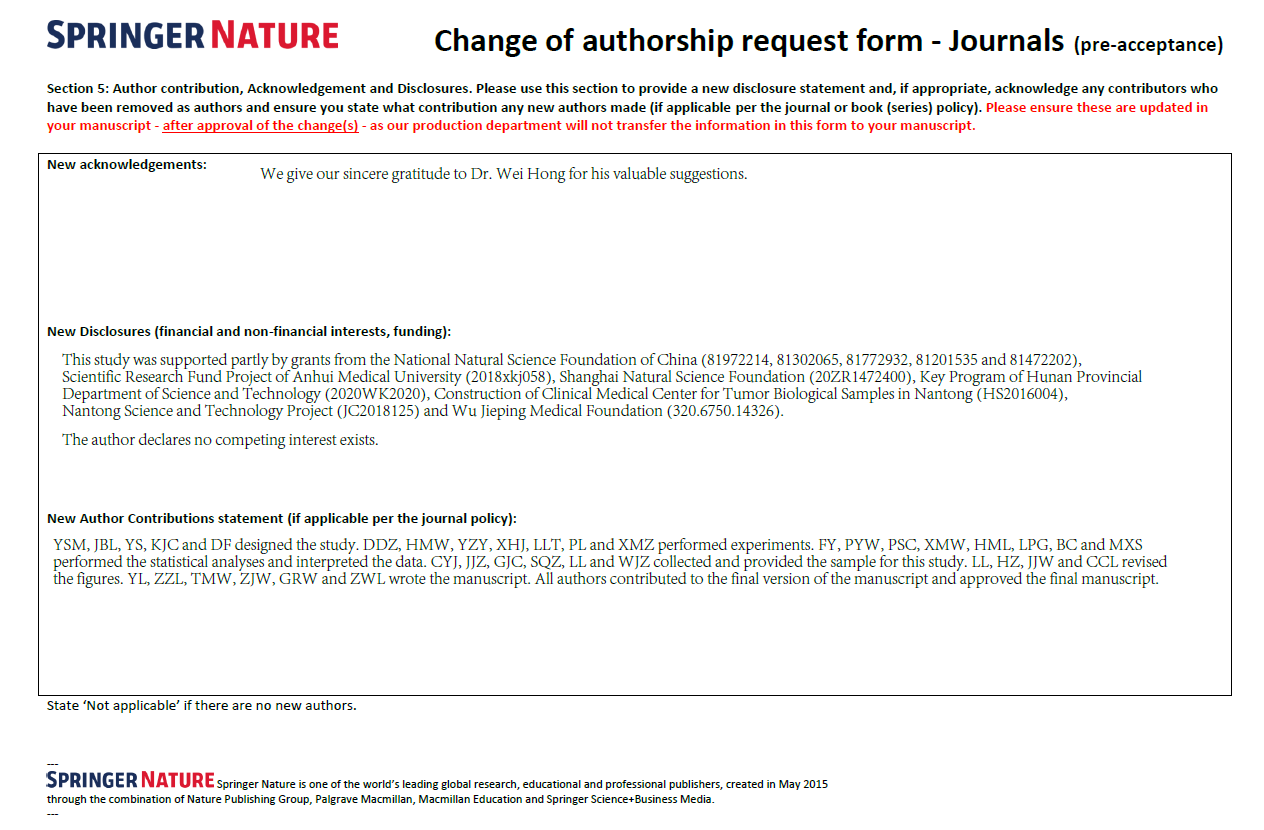


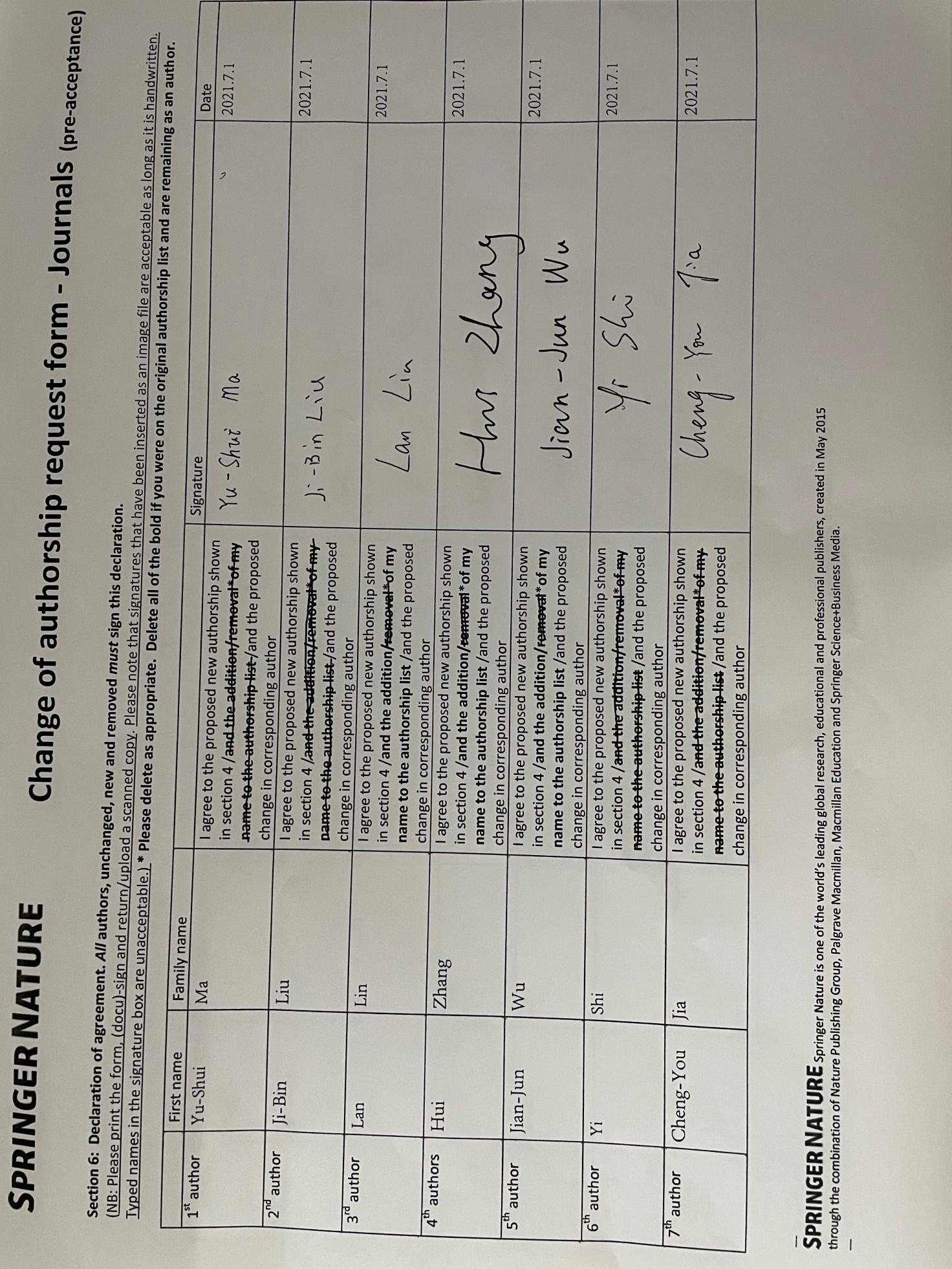


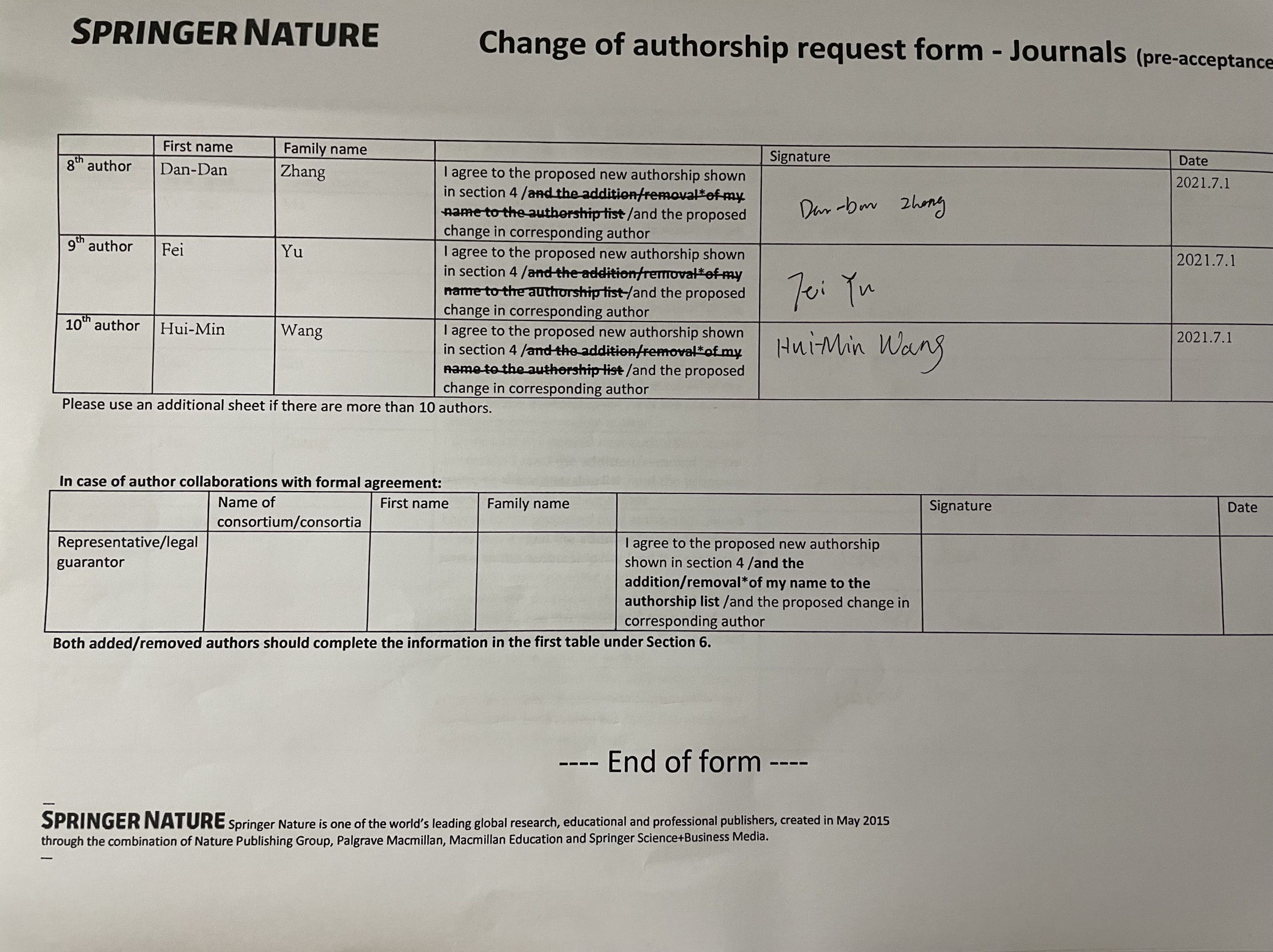


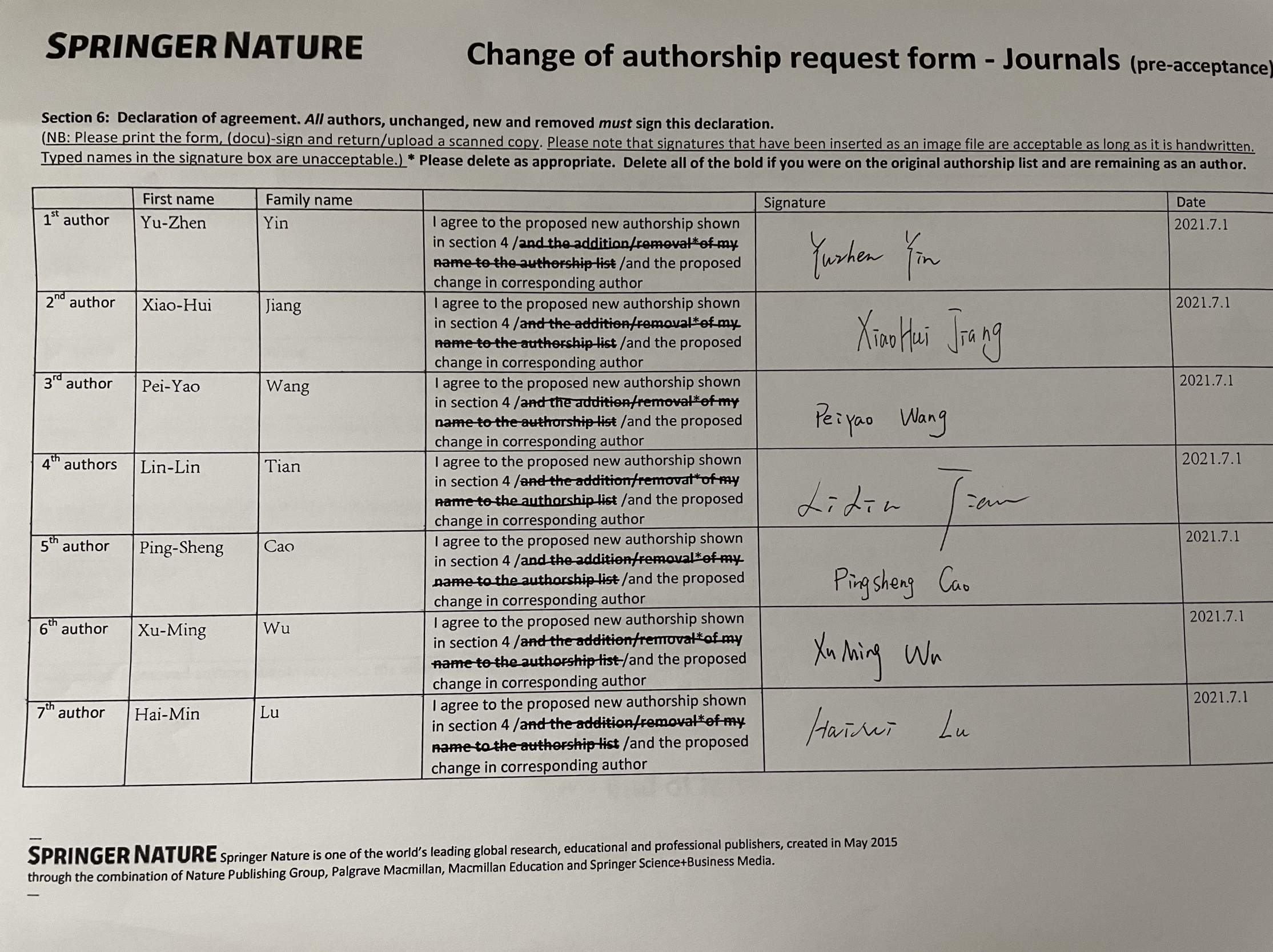


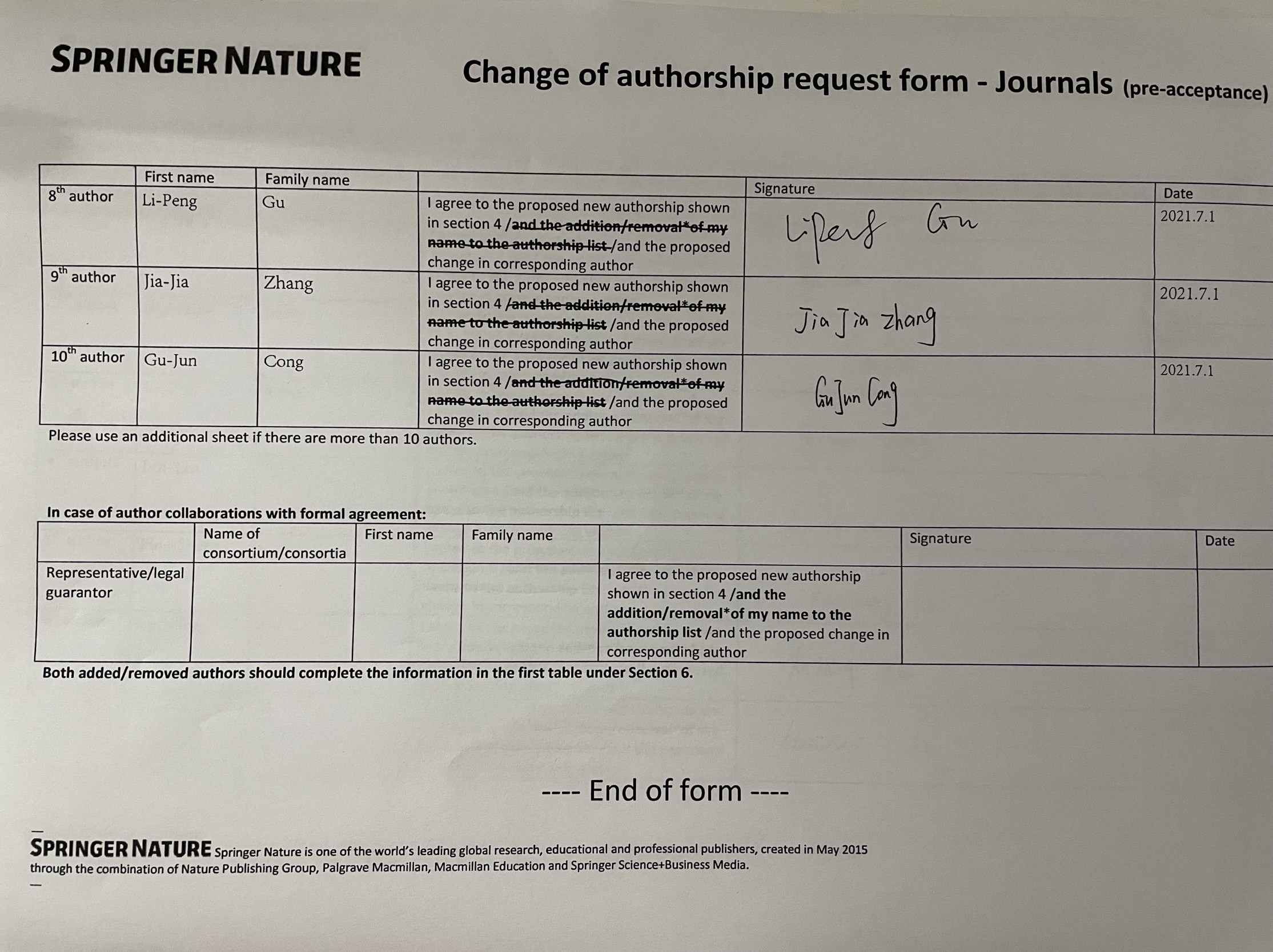


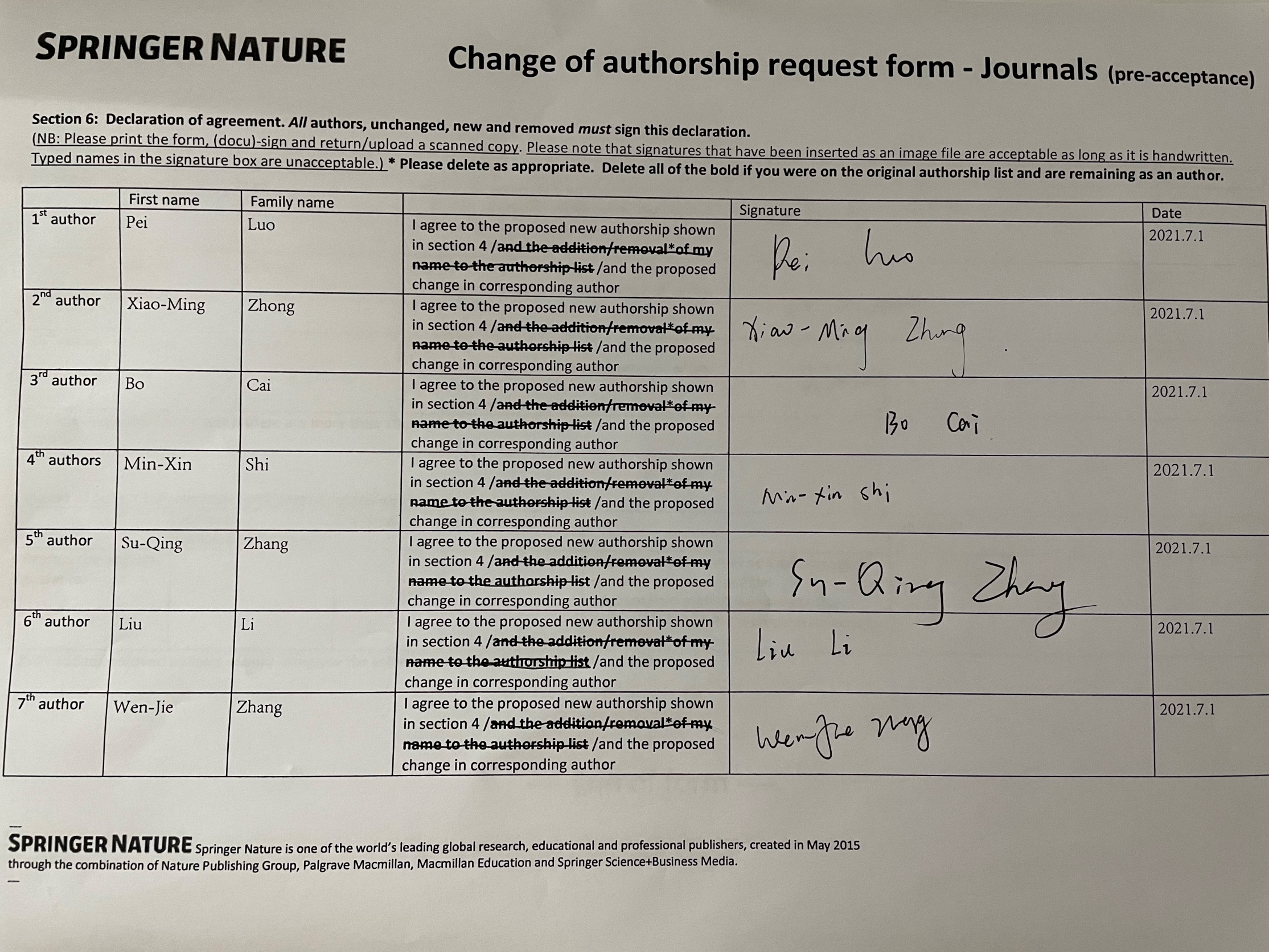


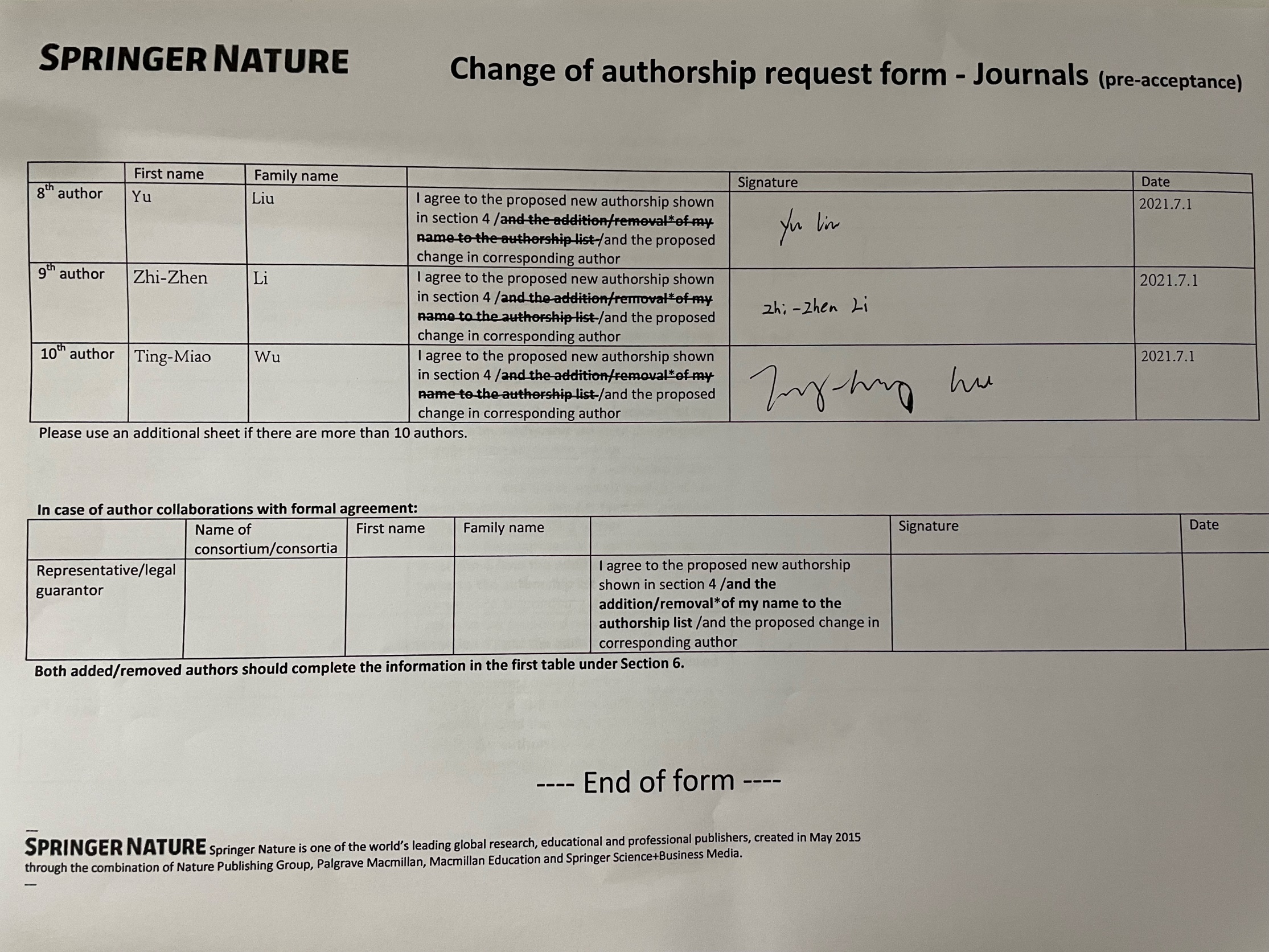


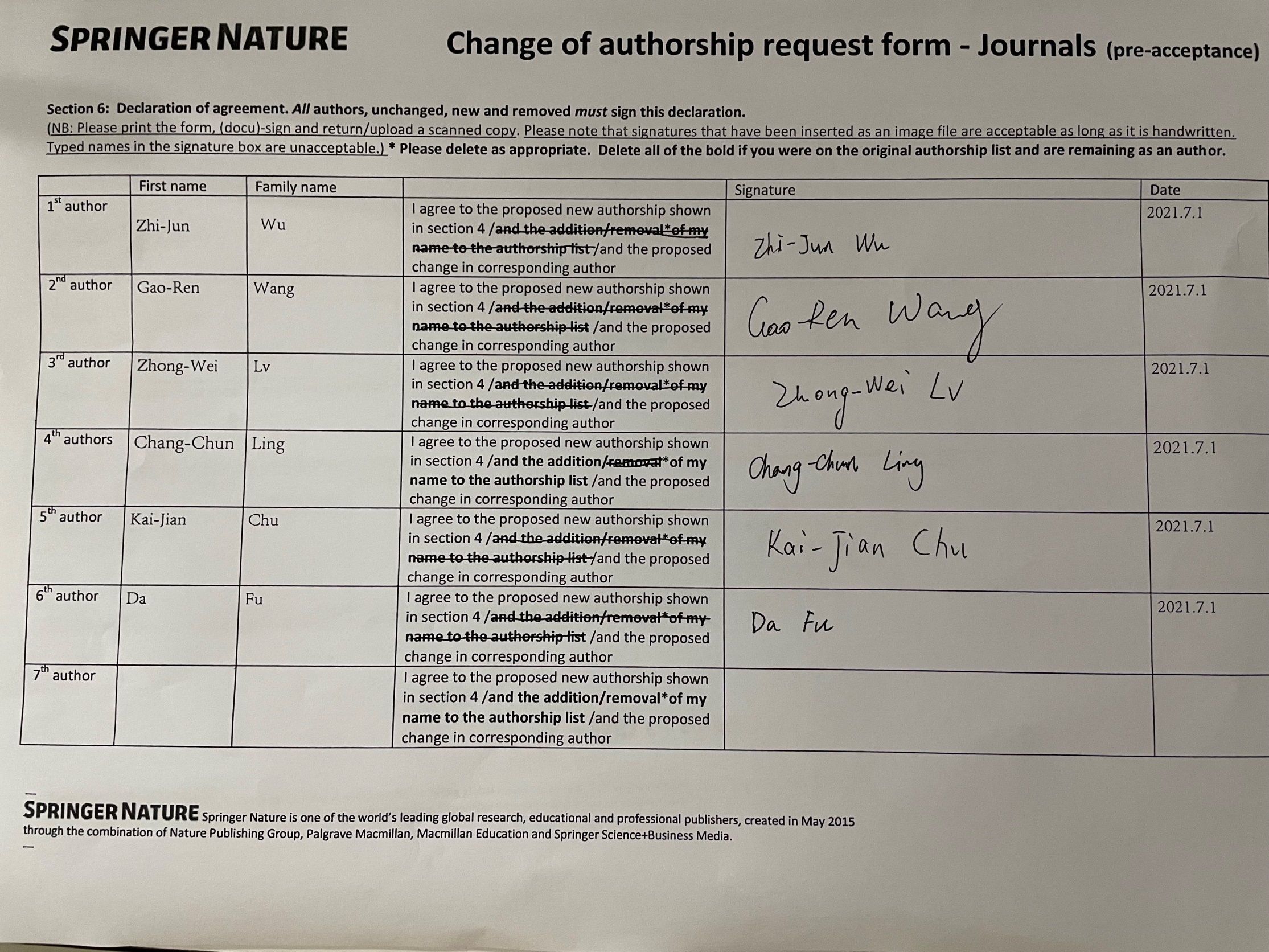


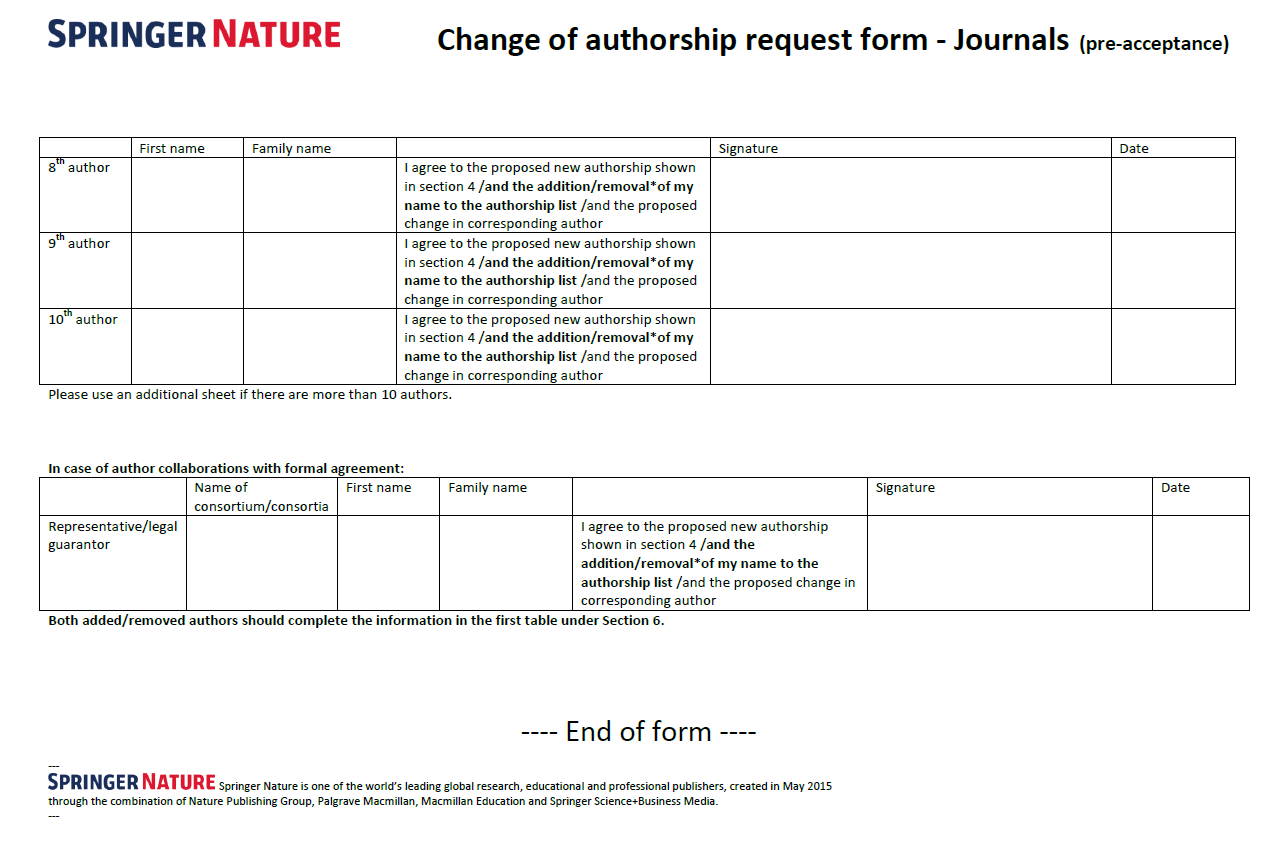

Supplement: Supplementary file 1 — Authorshipform_springer_nature [file 41420_2021_611_MOESM1_ESM.docx]

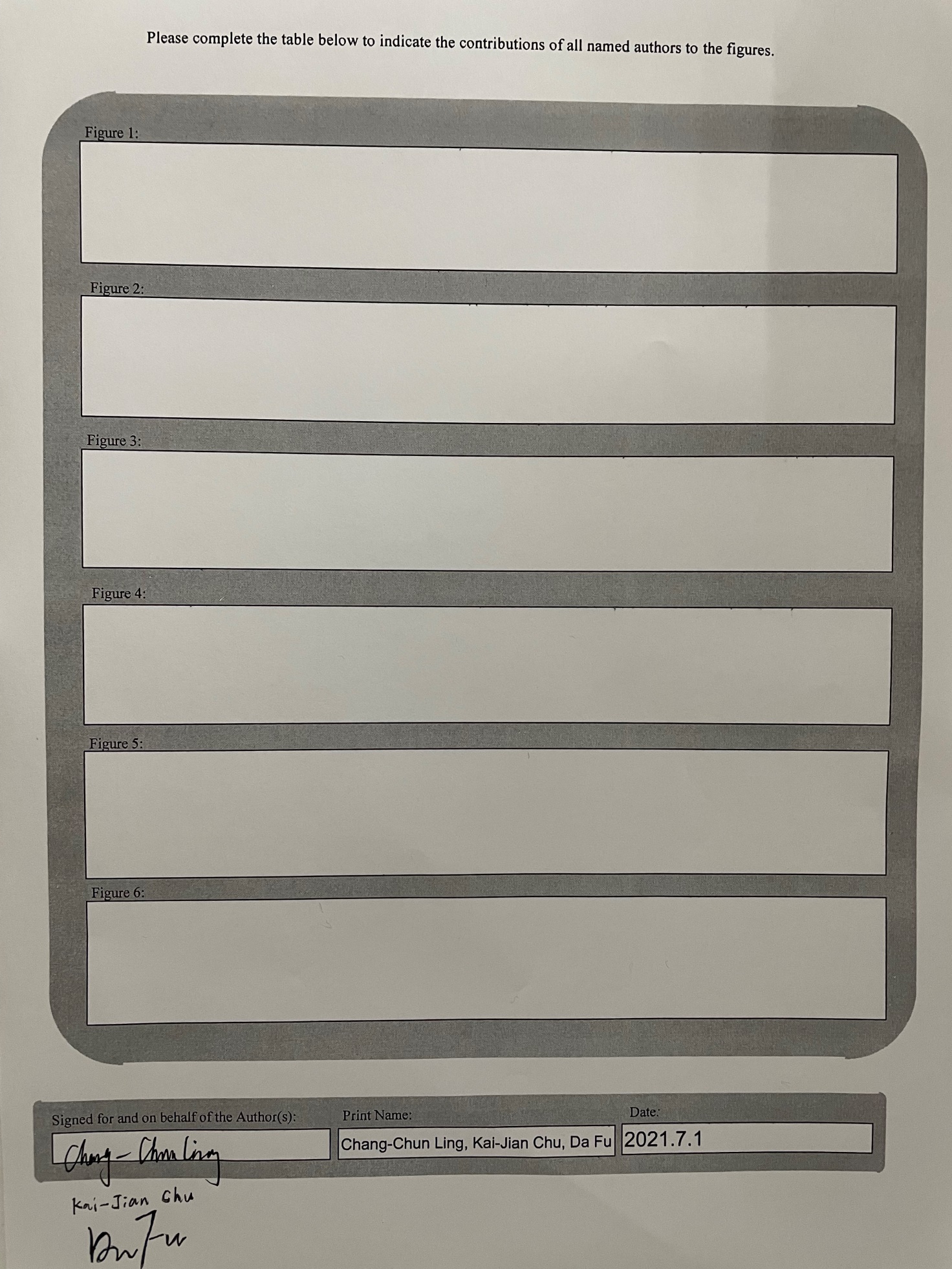

Supplement: Supplementary file 2 — cddiscovery-author-contribution-form [file 41420_2021_611_MOESM2_ESM.docx]
