# Supplementary material for: Exosomal microRNA-15a from mesenchymal stem cells impedes hepatocellular carcinoma progression via downregulation of SALL4
Source: Cell Death Discov. 2021 Aug 28;7:224. doi: 10.1038/s41420-021-00611-z (PMC8403170; doi:10.1038/s41420-021-00611-z)
Supplement: Supplementary file 3 — Table S1 [file 41420_2021_611_MOESM3_ESM.docx]

**Supplementary Table 1** Primer sequence for RT-qPCR

| Target | Sequences |
| --- | --- |
| miR-15a | 5’-GGGTAGCAGCACATAATGGT-3’ |
| U6 | 5’-CTCGCTTCGGCAGCACA-3’ |
| SALL4 | 5’-TGCAGCAGTTGGTGGAGAAC-3’ |
|  | 5’-TCGGTGGCAAATGAGACATTC-3’ |
| GAPDH | 5’-CTGGGCTACACTGAGCACC-3’ |
|  | 5’-AAGTG GTCGTTGAGGGCAATG-3’ |

Note: miR-15a, microRNA-15a; SALL4, spalt-like transcription factor 4; GAPDH, glyceraldehyde-3-phosphate dehydrogenase; RT-qPCR, reverse transcription quantitative polymerase chain reaction.
